# Intratumoral expression of IL-12 from lentiviral or RNA vectors acts synergistically with TLR4 agonist (GLA) to generate anti-tumor immunological memory

**DOI:** 10.1371/journal.pone.0259301

**Published:** 2021-12-02

**Authors:** Jardin A. Leleux, Tina C. Albershardt, Rebecca Reeves, Reice James, Jordan Krull, Andrea J. Parsons, Jan ter Meulen, Peter Berglund

**Affiliations:** Immune Design Corp., Seattle, WA, A wholly owned subsidiary of Merck & Co., Inc., Kenilworth, NJ, United States of America; Rutgers University, UNITED STATES

## Abstract

Systemic interleukin-12 (IL12) anti-tumor therapy is highly potent but has had limited utility in the clinic due to severe toxicity. Here, we present two IL12-expressing vector platforms, both of which can overcome the deficiencies of previous systemic IL12 therapies: 1) an integrating lentiviral vector, and 2) a self-replicating messenger RNA formulated with polyethyleneimine. Intratumoral administration of either IL12 vector platform resulted in recruitment of immune cells, including effector T cells and dendritic cells, and the complete remission of established tumors in multiple murine models. Furthermore, concurrent intratumoral administration of the synthetic TLR4 agonist **g**lucopyranosyl **l**ipid **A** formulated in a **s**table **e**mulsion (GLA-SE) induced systemic memory T cell responses that mediated complete protection against tumor rechallenge in all survivor mice (8/8 rechallenged mice), whereas only 2/6 total rechallenged mice treated with intratrumoral IL12 monotherapy rejected the rechallenge. Taken together, expression of vectorized IL12 in combination with a TLR4 agonist represents a varied approach to broaden the applicability of intratumoral immune therapies of solid tumors.

## Introduction

Interleukin-12 (IL-12) is an immunostimulatory cytokine produced mainly by antigen presenting cells after recognition of microbial danger signals, such as toll-like receptor (TLR) engagement [1, 2]. Upon secretion, IL-12 promotes several innate and adaptive immune cascades including natural killer (NK) cell activation and metastasis control [3, 4], macrophage and myeloid suppressor cell reprogramming [5–7], type I helper T cell differentiation [8, 9], CD8 T cell proliferation [10] and production of type II interferons [11–13].

While all these mechanisms provide immunological protection against cancer progression and metastasis, systemic administration of recombinant IL-12 protein can be accompanied by life-threatening adverse events related to overstimulated immune cells, such as cytokine release syndrome [14], effectively halting the use of systemic IL-12 therapy [15].

In order to make IL-12 immunotherapies safer and more targeted, various strategies have focused on delivering IL-12 locally to the tumor microenvironment (TME) to limit off-target toxicity. While local administration of recombinant IL-12 protein into injectable tumors results in less systemic toxicity, it also quickly diffuses away from the tumor microenvironment, limiting its therapeutic window [16]. Alternative methods of local delivery of IL-12 are being explored, including gene therapy.

Non-viral gene vectors, such as plasmid DNA and messenger RNA (mRNA), have been pursued as a potential solution to achieving controlled local expression of IL-12 while also offering modularity, ease of customization, and low inherent immunogenicity. IL-12 expression using these vectors has shown preclinical efficacy in various models, including immune-mediated regression of non-injected lesions (abscopal effects) [17–20]. However, success has been marginal in clinical trials conducted to date in melanoma and Merkel cell carcinoma patients, as treatment has not resulted in sustained anti-tumor responses or strong abscopal effects [21–23]. Others have developed mRNA vector platforms for local, transient expression of anti-tumor cytokines that are also currently being tested in the clinic [24].

Viral vectors engineered to express IL-12 have also been explored. Adenoviral vectors have broad tropism and provide the machinery for efficient delivery of transgenes, but their high expression profiles raise concerns around adverse toxic events similar to those observed after delivery of systemic recombinant IL-12 [25]. A solution currently being tested in the clinic is a chemically-triggered gene switch, which has shown promise in mice [26], as well as immune cell infiltration in a Phase I clinical trial [27]. However, this complicates the regimen significantly and may require careful calibration. Additionally, adenovectors are also susceptible to anti-vector immunity that may affect dosing consistency. Other vectors, such as alphavirus vectors, have shown preclinical promise but have not been evaluated clinically [28, 29].

Lentiviral vectors (LVs) are also attractive vehicles for gene delivery, particularly with the development of third generation LVs which addressed previous safety concerns while providing enhanced transduction efficiency, helping to increase therapeutic benefit overall [30]. We have developed a third generation lentiviral vector (ZVex^®^) for in vivo gene delivery to dendritic cells to induce antigen specific immune responses in cancer patients with an excellent safety profile and promising clinical benefit observed in a phase 1 trial [31, 32].

It is clear from these examples that there are two primary objectives that drive anti-tumor IL-12 gene therapy success: balancing efficacy and toxicity and driving a robust and long-lasting immunological response. Understanding and tuning the magnitude and kinetics of IL-12 expression is essential to achieve maximal therapeutic benefit. Here, we have developed lentiviral as well as *in vitro* transcribed alphaviral RNA vectors that can both be administered intratumorally to deliver IL-12 locally into the TME, providing the signals necessary to recruit and stimulate anti-tumor immune cell populations. Each vector induces extended production of IL-12, though the magnitude of the expression is distinct, allowing us to study IL-12 expression profiles that promote an anti-tumor response.

It has been published previously that IL-12 can drive superior effector T cell function, potentially at the cost of suboptimal central memory T cell development [33–35]. It has also been recognized that innate immunity drives much of the programming that dictates T cell fate [36]. In this study, we have combined our IL-12 expression vectors with an orthogonal approach of stimulating the innate immune system, namely the TLR4 agonist glucopyranosyl lipid A in a soluble emulsion (GLA-SE). These data represent improvements to the previously published IL-12 expression or delivery platforms.

## Methods

### Ethics statement

All procedures conducted with live vertebrate (mice) were approved by the Infectious Disease Research Institute Institutional Animal Care and Use Committee and all methods were carried out in accordance with relevant guidelines and regulations. Furthermore, each study was in compliance with ARRIVE guidelines including: all experiments included proper control groups, sample size for each experiment is described (S2 Table or figure legends), animals were randomized prior to treatment. Studies were not blinded.

### Integrating and non-integrating viral-vector design and production

Murine IL-12-expressing third-generation LVs targeting DC-SIGN via a modified Sindbis viral envelope were produced as previously described [37]. Integration deficient LVs included a class I mutation in the integrase gene, rendering it unable to insert its viral genome into the host cell [38]. LVs were quantified using a qPCR-based method described previously [39].

### RNA transcription and capping

Plasmids encoding self-replicating mRNA (srRNA) based on a modified Venezuelan Equine Encephalitis alphavirus (VEE, TC-83 strain [40]) were constructed by inserting the gene for murine IL-12 downstream of the VEE subgenomic promoter in place of the VEE structural polyprotein open reading frame. Self-replicating mRNA was produced using standard in vitro run-off mRNA transcription (MegaScript T7, ThermoFisher, Waltham, MA) and post-transcription capping protocols (Cap1 Kit, CellScript, Madison, WI), following by LiCl purification [41].

### GLA-SE formulation

The TLR4 agonist Glucopyranosyl Lipid A (GLA), formulated in a stable, 10% squalene oil-in-water emulsion was manufactured and formulated by Immune Design using proprietary methods. GLA-SE refers to formulation that has been diluted to 2% stable oil-in-water emulsion with HBSS.

### RNA formulation

srRNA was formulated using In Vivo jetPEI (Polyplus, Strasbourg, France) according to the manufacturer’s instructions. Briefly, srRNA was mixed gently with jetPEI in a 5% glucose diluent and complexed by brief incubation at room temperature. The addition of GLA-SE, when applicable, always followed RNA complex formation.

### Animals

Female 7-week-old C57BL/6 or BALB/c mice were purchased from the Jackson Laboratory (Bar Harbor, ME) and housed in a BSL2+ level room under reduced-pathogen conditions. Husbandry and facilities were maintained by the Infectious Disease Research Institute (Seattle, WA).

### Tumor studies

For tumor implantation, mice were anesthetized and inoculated with syngeneic cancer cell lines. For the B16 melanoma model, C57BL/6 mice were inoculated subcutaneously either in the flank or footpad with 1x10^5^ or 1x10^6^ B16-F10 murine melanoma cells, respectively. GL261 glioma cells were implanted subcutaneously into the flank of C57BL/6 mice. Female BALB/c mice were inoculated subcutaneously in the flank with 1x10^5^ CT26 colon carcinoma, 5x10^6^ A20 B cell lymphoma or 1x10^4^ P815 mastocytoma cells. Alternatively, BALB/c mice were inoculated in their mammary fat pad with 1x10^5^ 4T1 mammary carcinoma cells.

### Intratumoral injections

Intratumoral injections were performed once tumors were palpable, usually 7–10 days following implantation. Prior to intratumoral injection, LVs and formulated RNA were diluted to a total volume of 50 μL/injection in cold HBSS or 5% glucose, respectively, and kept on ice. srRNA/mIL12 were administered once weekly, while LVs were only administered once. When GLA-SE was included in the treatment regimen, it was diluted to 2% stable emulsion prior to the injection and administered biweekly starting 24 hours after the single injection of LV/mIL12 or concomitantly with formulated srRNA/mIL12.

### Monitoring of IL-12 or IFNγ in serum

In-life monitoring of serum content of IL-12 or IFNγ was conducted by retro-orbital collection of whole blood into serum collection tubes (BD Biosciences, Waltham, MA), not exceeding 10% of the mouse total blood volume. Whole blood was then centrifuged to remove cells and serum was collected and stored at -20°C until analysis. ELISAs for detection of IL-12 and IFNγ were used for quantification (ThermoFisher 88-7121-22 and 88-7314-22, LLOQ = 4 pg/ml).

### Tumor isolation for flow cytometry

Following flank inoculation of B16 melanoma tumors, mice were either treated or left untreated (control mice). Around 18 days post-inoculation, mice were euthanized and tumors were isolated using scissors and forceps and dissociated using a GentleMACS (Miltenyi, Bergisch Gladbach, Germany). Tumor cell suspensions were then added to a Ficoll gradient using a SepMate tube system (StemCell Technologies, Vancouver, CA) and centrifuged for 10 minutes at 1200 x *g*. Tumor cells were pelleted, while infiltrating immune populations were isolated in the top layer of the gradient. These cells were washed to remove Ficoll, then stained using a combination of FITC-Ly6G, FITC-NKp46, PE-CD11b, PE-Foxp3, PerCP-Cy5.5 F480, PerCP-Cy5.5 CD3e, APC-Ly6C, APC-CD25, AlexaFluor700-CD4, eFluor-450-CD8, PacBlue-CD11c, BV500-B220 or BV500-CD8a and Live Dead near-IR stain (Biolegend, San Diego, CA or ThermoFisher, Waltham, MA).

### Tumor isolation for RNA transcriptomics and Nanostring analysis

Around 18 days post-inoculation, treated or untreated B16 melanoma tumors were removed from sacrificed mice using scissors and forceps and dissociated using the RNA isolation setting of a GentleMACS (Miltenyi, Bergisch Gladbach, Germany). RNA was isolated from tumor lysates using a RNeasy Mini Kit (Qiagen, Hilden, Germany) and Nanostring analysis using the murine PanCancer Immune Oncology panel of 770 genes was performed. Data analysis was performed using the Advanced Analysis tool of nSolver. Gene expression changes were scored in comparison to the mean expression across the entire experiment. From there, gene scores were batched by function or cell phenotype. Gene score batches were then clustered hierarchically based on the degree to which the genes changed.

### IVIS imaging

Mice were injected with 150 mg/kg D-luciferin (Perkin Elmer, Waltham, MA) dissolved in HBSS, intraperitoneally. Mice were then imaged using an IVIS system 15–20 minutes following injection.

### Statistical analysis

ANOVA tests with Tukey’s test for multiple comparisons were used to determine statistical significance and performed using Prism 8.0 (GraphPad, La Jolla, CA). Survival statistical significance was determined using a Log-rank test. *P* values equal to or less than 0.05 were considered significant. Results of statistical analyses are reported in S2 Table.

## Results

### Transduction with ILVs results in higher expression of encoded transgene than NILVs

**Fig 1:**
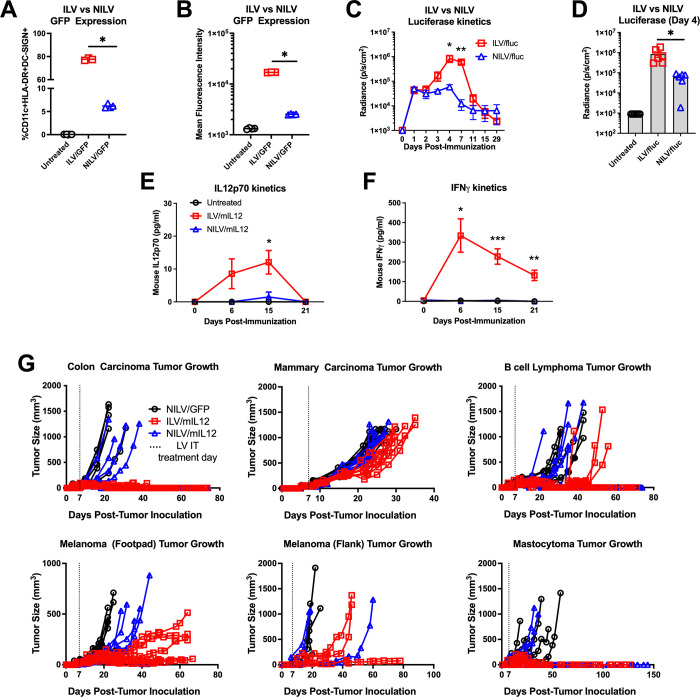
Integrating lentiviral vectors induce greater IL12 expression, leading to superior control in multiple syngeneic tumor models. **(A, B)** CD11c+ HLA-DR+ DC-SIGN+ human dendritic cells were transduced with integrating lentiviral vector (ILV) or non-integrating lentiviral vector (NILV) expressing GFP for 72 hours. (*p* < 0.0001, representative data from 3 independent experiments). **(C, D)** Mice were injected with ILVs or NILVs expressing firefly luciferase (1 x 10^10^ vector genomes) subcutaneously at the base of the tail on Day 0. Luciferase expression was monitored over 28 days (*p = 0.006, **p < 0.0001 representative data from 3 independent experiments). **(E, F)** Mice inoculated with B16F10 syngeneic melanoma tumors in their flanks were treated with a single shot of NILVs or ILVs expressing mouse IL12 on Day 0. Whole blood was collected from mice weekly until 21 days post-immunization and serum was analyzed for IL12p70 (*p = 0.02) and IFNγ (*p = 0.004, **p = 0.0005, ***p < 0.0001, representative data from 3 independent studies). **(G)** Mice were inoculated subcutaneously with colon carcinoma (CT26, n = 9), B cell lymphoma (A20, n = 9), melanoma (B16, n = 3–8) or mastocytoma (P815, n = 5) or orthotopically with mammary carcinoma (4T1, n = 9), then treated with a single shot of either ILV expressing mIL-12 or NILV expressing mIL-12 when tumors were palpable (Day 7–10). Graphs represent tumor growth of individual mice in each treatment group. Data points and error bars in **(A-F)** represent mean and standard error of the mean for representative studies, respectively. Statistical significance was determine using one-way ANOVA analysis followed by Tukey multiple comparison tests and *p* < 0.05 was considered significant.

Third-generation integrating and non-integrating LVs (ILVs and NILVs, respectively) were previously designed to transduce human dendritic cells via binding to DC-SIGN (DEC-209) [38, 39]. ILV and NILV mediated transgene expression magnitude and kinetics were assessed both in vitro and in vivo using expression of GFP and luciferase, respectively. Transduction of human dendritic cells (CD11c+HLA-DR+DC-SIGN+) with NILVs resulted in fewer cells expressing detectable levels of GFP than cells transduced with ILVs (Fig 1A, *p* < 0.0001, S1A Fig). Additionally, the GFP+ populations expressed less GFP per cell when transduced with NILVs in comparison to ILVs (Fig 1B, *p* < 0.0001). The same finding was observed in vivo with luciferase-expressing LVs, indicating that these vectors successfully transduce murine cells as well. While the kinetics of *luc* expression did not differ much between NILVs and ILVs (Fig 1C, *p = 0.006, **p < 0.0001), administration of ILV mediated significantly higher transgene expression levels in vivo (Fig 1D, *p* = 0.006, S1B Fig).

NILVs and ILVs were similarly evaluated for their ability to mediate expression of murine IL-12 (mIL-12) in vivo using a quantitative ELSA specific for the active heterodimer of IL-12, IL12p70. Following intratumoral administration, levels of IL12p70 found in the serum were detectable 6 days after injection with ILVs only and peaked at approximately the 2-week time point. Circulating IL12p70 was no longer detectable 3 weeks after injection (Fig 1E, *p = 0.02). Mice with tumors injected with NILV had serum levels of IL-12 below the limit of detection, consistent with lower luciferase expression levels described above. Elevated serum levels of endogenous interferon-γ (IFNγ) correlated with induction of mIL-12, consistent with biological activity of IL-12 (Fig 1F, *p = 0.02, **p = 0.001, ***p < 0.0001).

### Intratumoral administration of ILV/mIL12 provides significant survival benefit in several syngeneic tumor models

Several syngeneic murine tumor models were used to assess the potential anti-tumor effect of ILV/mIL12 (Fig 1G). Tumors from a variety of histological origins were implanted subcutaneously, including colon carcinoma (CT26), B cell lymphoma (A20), melanoma (B16) and mastocytoma (P815). A mammary carcinoma (4T1) was tested orthotopically where tumors were implanted into the mammary fat pad. Additionally, a glioma model (GL261) was tested non-orthotopically where tumors were implanted subcutaneously into the mouse flank (S1D Fig). Once tumors were palpable, mice received a single intratumoral injection of either NILVs encoding mIL12 (NILV/mIL12), ILVs encoding mIL-12 (ILV/mIL12) or a control lentiviral vector encoding GFP intratumorally. In all cases, mice that were treated with ILV/mIL12 experienced a delay in tumor growth and, in many cases, complete tumor regression. This led to enhanced survival across all models (S1C Fig, S1 Table). NILV/mIL12 treatment provided mice with minimal protection in some models but was not as potent as ILV/mIL12 likely because IL-12 expression from these vectors was below the level of therapeutic impact. Based on the above findings, when the same number of vector genomes was used, compared to NILV, ILV transduced a greater number of cells and induced greater gene expression (quantified by GFP or luciferase expression). When mIL-12 was expressed, ILV led to greater anti-tumor control. For these reasons, we conclude ILV to be more efficacious than NILV, and thus, subsequent investigations were conducted using ILV/mIL12.

**Fig 2:**
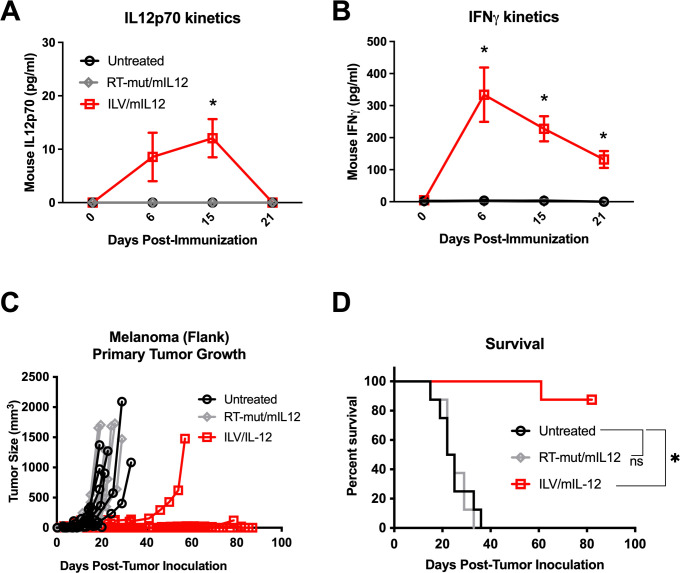
Tumor control following ILV immunization is mediated by IL12. **(A, B)** Mice inoculated with B16 melanoma tumors in their flanks (1E5 cells) were treated with a single shot of ILV/mIL12 (1.5E10 vgs) with or without a mutation in reverse transcriptase machinery (“RT-mut”) that were not capable of expressing the transgene on Day 7. Whole blood was collected retro-orbitally from mice weekly until 21 days post-immunization. Serum was isolated and analyzed for IL12p70 (*p = 0.005) and IFNγ (*p < 0.004, representative data from 3 independent experiments). These assays were run simultaneously with the samples from Fig 1E and 1F and therefore share data for untreated and ILV/mIL12 groups. **(C, D)** Mice inoculated with B16 melanoma tumors in their flanks were treated with a single shot of ILV/mIL12 or RTmut/mIL12 on Day 7 and tumor growth was monitored (*p* < 0.0001, n = 8, 1 independent study). Data points and error bars in **A, B** represent mean and standard error of the mean, respectively. Statistical significance was determine using one-way ANOVA analysis followed by Tukey multiple comparison tests and *p* < 0.05 was considered significant. Tumor growth plots represent individual mice. Survival benefit was determined using Mantel Cox Log-rank testing where *p* < 0.05 is considered significant.

To confirm that the observed therapeutic benefit was mediated by the in vivo expression of mIL-12 and not by mIL-12 present in the ILV vector preparation (ILV producer cells are transfected with mIL-12 plasmid) or by a general innate immune response generated following lentiviral vector transduction, we designed an ILV/mIL12 vector with a lethal mutation in the reverse transcriptase machinery (RT-mut/mIL12), which prevents transgene expression in vivo, while maintaining all the viral component parts that could trigger an inflammatory response. To confirm the dysfunction of RT-mut ILVs, mice were immunized intratumorally with either the wild type or mutated ILVs intratumorally and serum was collected at multiple timepoints for mIL-12 quantification (Fig 2A, *p = 0.02). No detectable IL12p70 was observed and endogenous IFNγ was not produced at levels that were detectable by ELISA (Fig 2B). Mice bearing subcutaneous B16 melanoma tumors were injected intratumorally with either wild-type or mutated ILV/mIL12 and tumor growth was monitored (Fig 2C). Mice receiving functional ILV/mIL12 rejected their tumors which resulted in 90% overall survival (Fig 2D, *p = 0.0002), while those receiving mutated ILV/mIL12 saw no therapeutic benefit of the lentiviral immunization. Similar activity was observed in other tumor models as well (S2A and S2B Fig). These data validate that ILV/mIL12-mediated local production of IL12p70 in the TME is responsible for the observed therapeutic effect.

### Intratumoral ILV/mIL12 treatment provides anti-tumor protection against injected and non-injected lesions

**Fig 3:**
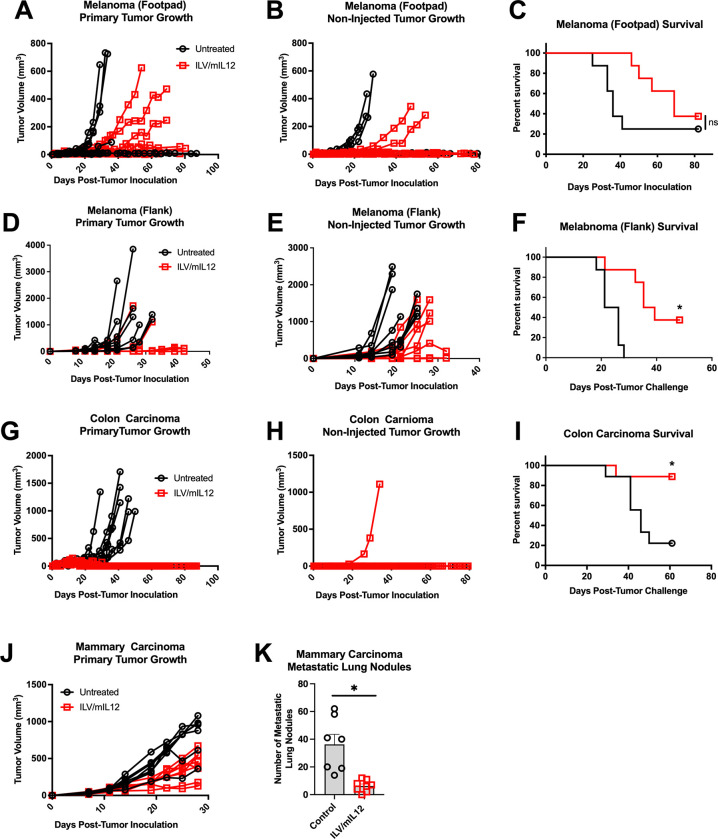
ILV/mIL12 provides protection against distant, non-injected tumors. Mice were inoculated with **(A-C)** B16 melanoma tumors in both of their footpads (1E6 cells, n = 8), (**D-F)** B16 melanoma tumors subcutaneously in both of their flanks (1E5 cells, n = 10) or **(G-I)** CT26 colon carcinoma tumors subcutaneously in both of their flanks (1E5 cells, n = 9), then treated with ILV/mIL-12 in the right flank tumor when tumors were palpable (Day 7–10), leaving the left flank tumor untreated (**F,** *p* < 0.0001; **J**, *p* < 0.02; representative data from 5 independent experiments) **(J, K)** Mice were inoculated orthotopically with a single 4T1 mammary carcinoma tumor their mammary fat pad, then treated with ILV/mIL12 intratumorally (n = 8). Mouse lungs were isolated to count metastatic lung nodules 18 days after inoculation (*p* = 0.0008, representative data from 3 independent experiments). Tumor growth plots represent individual tumors on each mouse. Survival benefit was determined using Mantel-Cox Log-rank testing where *p* < 0.05 is considered significant. Data points and error bars in K represent mean and standard error of the mean, respectively. A Student’s T-test was used to determine significance.

To investigate whether intratumoral treatment would induce systemic immune responses impacting non-injected lesions (abscopal treatment effect), several bilateral tumor models were developed and tested. For the subcutaneous models (i.e. melanoma and colon carcinoma), tumors were implanted bilaterally, and only one tumor received ILV/mIL12 treatment. For all models, intratumoral treatment of a primary tumor with ILV/mIL12 led to delayed tumor growth of the non-injected lesion and increased survival (Fig 3). Specifically, about 40% of animals bearing B16 melanoma tumors (either in their footpads or flanks) were able to survive bilateral tumor challenge after only one injection of ILV/mIL12 in the primary tumor (Fig 3A–3F, *p* < 0.0001). Survival was just under 90% for mice challenged with CT26 colon carcinoma tumors (Fig 3G–3I, *p* = 0.009).

In order to assess if the ILV/IL12-mediated therapeutic effect on distal tumors also would translate into an effect on metastatic lesions, we set up a naturally metastatic mammary carcinoma model (4T1) in which tumors were implanted in the mammary fat pad, which led to the spontaneous formation of tumor nodules in the lungs. Mice were injected with ILV/mIL12 into the primary tumor, and after 18 days, mice were sacrificed for lung nodules enumeration. Mice bearing 4T1 mammary carcinoma tumors had 6 times fewer metastatic lung nodules after treatment with ILV/mIL12 compared to untreated controls (Fig 3J, 3K, *p* = 0.0003). These data demonstrate that local administration of IL-12 could lead to generation of systemic anti-tumor response capable of controlling both local (treated) and distant (non-treated) tumor growth.

### IL-12-induced tumor elimination requires CD8 T cells

**Fig 4:**
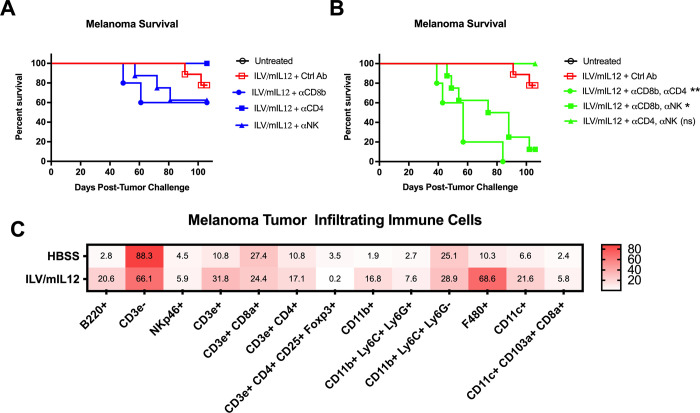
CD8 T cells are critical for IL12 mediated tumor elimination. **(A)** Mice inoculated subcutaneously with B16 melanoma tumors in their right flanks (n = 8–9) were depleted of one or **(B)** two critical immune populations using antibodies against CD8α, CD4 or NK1.1 (to deplete CD8 T cells, CD4 T cells or natural killer cells, respectively) throughout the experiment (n = 5–10). Mice were then given a single intratumoral injection of ILV/mIL12 on Day 7 and tumor growth was monitored. Survival benefit was determined using Mantel-Cox Log-rank testing where *p* < 0.05 is considered significant (*p = 0.002, **p = 0.0001). **(C)** Mice were inoculated with subcutaneous B16 melanoma tumors in their right flanks and then received one shot of ILV/mIL12 (n = 5). Tumors were isolated 7 days after ILV administration and infiltrating immune cells were isolated and phenotyped by flow cytometry analysis. Numbers in each square represent the percentage stained cells of live cells found in TIL population.

IL-12 is a potent activating and proliferative factor for not only T cells but also natural killer (NK) cells [3, 4]. It promotes the differentiation of naïve CD4 T cells towards type 1 helper status and enhances the cytotoxicity of CD8 T cells and NK cells [42]. To identify which of these subsets mediates the antitumor activity of ILV/mIL12, we systematically depleted individual or multiple effector cell types in vivo in treated mice. Surprisingly, depletion of either CD8, CD4 or NK cells, only marginally diminished the therapeutic effects, suggesting redundant mechanisms (Fig 4A). We therefore performed systematic depletions of combinations of lymphocyte subsets. Depletion of CD8 T cells simultaneously with another effector population (either CD4 T cells or NK cells) led to a significant reduction in survival benefit of ILV/mIL12 treatment (Fig 4B, *p = 0.002, **p = 0.0001, S3 Fig). This was not observed in mice that were depleted of CD4 and NK cells, but had an intact CD8 T cell compartment, indicating that CD8 T cells play an important role in the success of IL-12-mediated anti-tumor protection. Taken together, these observations potentially point to an interplay between CD4 T cells (potentially regulatory T cells) and effector cells (i.e., CD8 T cells and NK cells) that emphasizes the importance of CD8 T cell activity as the main mediator of IL-12 anti-tumor activity.

To determine whether the composition of other innate and adaptive immune cells changes in the TME after intratumoral injection of ILV/mIL12, B16 melanoma tumors were isolated and infiltrating immune cell populations were phenotypically characterized by flow cytometry following treatment. Several cell populations increased in tumors treated with ILV/mIL12, notably B220+ B cells, CD3ε+ T cells, CD11b+ myeloid cells, F480+ macrophages, and CD11c+ dendritic cells including CD11c+CD103+ cross-presenting dendritic cells (Fig 4C). This is indicative of general immune activation and inflammation, which is consistent with previous reports describing the effects of local IL-12 therapy [43]. This hypothesis also offers an explanation for why CD8 T cells are not enriched in tumor following IL-12 treatment and points to a mechanism of action where activation state of the T cells are more important than quantity.

### Formulated self-replicating RNA is a simple and versatile platform for IL-12-mediated immunotherapy

**Fig 5:**
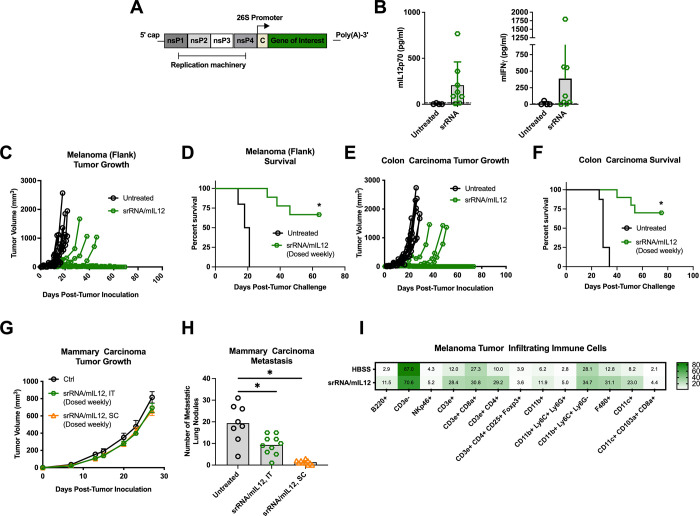
Self-replicating mRNA encoding mIL12 provides tumor control and immune cell infiltration. **(A)** Self-replicating messenger RNA (srRNA) includes machinery that allows for amplification of the transgene in the cytoplasm of transfected cells. **(B)** Mice inoculated with subcutaneous B16 melanoma tumors in their right flanks were treated with a single shot of polyethyleneimine-formulated-srRNA expressing mIL12 (srRNA/mIL12) on Day 7 and whole blood was collected retro-orbitally 3 days later. Serum was isolated and analyzed for IL12p70 and IFNγ (n = 4–8). Mice were inoculated subcutaneously with **(C, D)** B16 melanoma tumors (n = 9) or **(E, F)** CT26 colon carcinoma tumors in their right flanks and given weekly shots of polyethyleneimine-formulated-srRNA/mIL12 intratumorally (n = 8) (**D** & **F**, *p < 0.0001, representative data from 3 independent experiments). **(G)** Mice were inoculated orthotopically with 4T1 mammary carcinoma tumors in their mammary fat pads and given weekly shots of polyethyleneimine-formulated-srRNA/mIL12 either intratumorally or subcutaneously at their tail base. **(H)** Mice challenged with orthotopic 4T1 mammary carcinomas then treated with weekly injections of formulated srRNA/mIL12 either intratumorally or subcutaneously were sacrificed 18 days after tumor inoculation to evaluate metastatic lung nodule formation (IT, *p* < 0.002; SC, *p* < 0.0001; n = 8–10; representative data from 2 independent experiments). **(I)** Mice were inoculated subcutaneously with B16 melanoma tumors in their right flanks and then received weekly shots of polyethyleneimine-formulated-srRNA/mIL12. Tumors were isolated 25 days after inoculation and infiltrating immune cells were isolated and phenotyped by flow cytometry analysis (n = 5). Data points and error bars in **B** and **H** represent mean and standard error of the mean, respectively. Student’s T test was used to determine statistical significance with a 95% confidence interval. Tumor growth plots represent individual mice. Survival benefit was determined using Mantel-Cox Log-rank testing where *p* < 0.05 is considered significant. Numbers in each square represent the percentage stained cells of live cells found in tumor infiltrating lymphocyte population.

RNA vaccines have recently received significant attention due to their ease of scalable manufacture and potential for highly controlled dosing. We therefore evaluated a self-replicating messenger RNA (srRNA) platform for intratumoral IL-12 therapy. In addition to encoding for the IL-12 gene, srRNAs also encoded for their own replication machinery (Fig 5A) and therefore generated significantly higher levels of expression than traditional messenger RNA (S4A and S4B Fig, *p* = 0.02). Expression of the transgene is also high *in vivo* and can be detected for approximately two weeks after injection (S4C Fig). srRNAs, like most nucleic acid vectors, require specific formulations to improve their stability and delivery to cells after administration. Polyethyleneimine (PEI) is a commercially available cationic polymer that has been extensively studied in vaccinology as an adjuvanting delivery vehicle [44]. Mouse IL-12 encoded srRNA was therefore formulated with an *in vivo* grade PEI (srRNA/mIL12) to form transfection complexes for intratumoral administration.

srRNA/mIL12 immunization produced functional protein *in vivo*, which was confirmed by quantification of both mIL-12 and mIFNγ in the serum of immunized animals (Fig 5B). A single injection of srRNA/mIL12 provided a significant delay in tumor growth but ultimately all mice succumbed to their tumor burden, making this approach significantly less effective than ILV/mIL12 (S5A and S5B Fig, p = 0.009). Overall survival was improved when weekly doses of srRNA/mIL12 were administered (S5C and S5D Fig). Weekly intratumoral injections of srRNA/mIL12 were sufficient to eliminate 66% of B16 melanoma tumors and 70% of CT26 colon carcinoma tumors (Fig 5C–5F, *p* < 0.0001). Like ILV/mIL12 treatment, srRNA/mIL12 did not provide significant protection against primary mammary carcinoma tumors (Fig 5G) but did effectively minimize the growth of metastatic lung nodules (Fig 5H, *p* < 0.002).

To further assess the two platforms, we isolated infiltrating immune cell populations from B16 melanoma tumors treated with weekly immunizations of srRNA/mIL12 to determine cell composition of the TME. Similar to ILVs, srRNA/mIL12 promoted the migration and/or expansion of B220+ B cells, CD11b+ myeloid cells, F480+ macrophages and CD11c dendritic cells, as well as CD3ε+ T cells (Fig 5I). For T cells, particularly CD4 T cells were recruited/induced, but regulatory T cells were not observed.

### Durable anti-tumor response is achieved when intratumoral IL-12 is combined with GLA-SE

Successful immunotherapies aim to induce immune memory in addition to tumor control and elimination, aiding in prevention of relapse and control of micrometastases. While GLA-SE has been evaluated in various tumor models, we primarily used the B16F10 melanoma model because of its known T cell epitopes that could be used to track T cell responses post-treatment.

**Fig 6:**
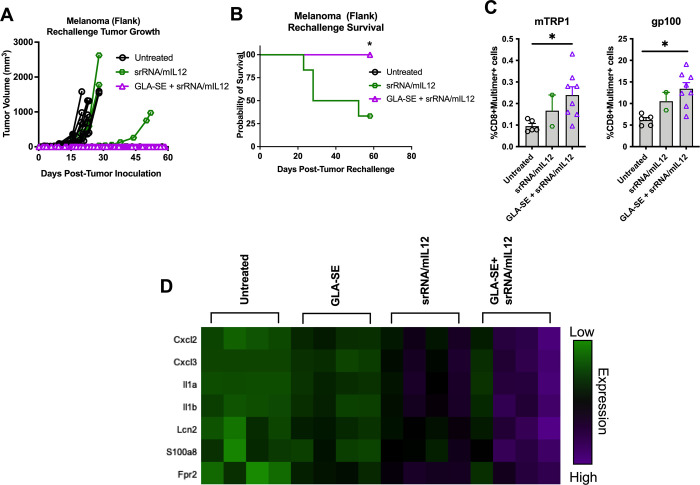
GLA-SE improves IL12 induced tumor control, antigen-specific T cell expansion and myeloid cell activation. **(A, B)** Mice bearing subcutaneous B16 melanoma tumors in their right flanks were treated initially with srRNA/mIL12 with or without the combination of the TLR4 agonist GLA-SE (n = 9–10). Mice that survived initial tumor challenge were rechallenged with a second identical tumor inoculation. Tumor growth was monitored with no further treatment (*p < 0.0001; representative data from 3 independent experiments). **(C)** Mice that survived until 66 days after rechallenge were sacrificed for splenocyte isolation. Splenocytes were stained for melanoma antigen-specific T cells populations (e.g. TRP1 and gp100) and analyzed using flow cytometry (*p* = 0.0456 and *p* = 0.005, respectively). **(D)** NanoString expression profiles of most upregulated genes in B16 tumors isolated from mice treated with srRNA/mIL12 with or without the combination of GLA-SE (n = 5). Tumor growth plots represent individual mice. One-way ANOVA analysis was used to determine significance of plots in **C.** Survival benefit was determined using Mantel-Cox Log-rank testing where *p* < 0.05 is considered significant. Gene analysis was conducted using the Advanced Analysis module of the nSolver software program.

To model tumor reoccurrence, mice that rejected their initial tumor burden following intratumoral mIL-12 treatment (either via ILV or srRNA) were rechallenged with the same parental tumor cell line subcutaneously in the opposite (untreated) flank. No treatments were subsequently administered, and mice were monitored to determine their ability to reject the tumor rechallenge. Surprisingly, most mice that received either ILV/mIL12 or srRNA/mIL12 treatments were not able to successfully mount a recall response that was sufficient for tumor protection (Fig 6A and 6B, *p = 0.007, S6A Fig).

It has previously been shown that low antigen expression, weak antigen-TCR interaction, and weak co-stimulation can reduce efficient memory T cell induction. However, strong pro-inflammatory cytokine signaling (signal 3). which can be provided by IL-12 and type 1 interferons enhanced the memory response in an infectious disease setting [45]. To this end, we investigated GLA-SE, which induces proinflammatory cytokines and type 1 interferons [46], together with srRNA expressing mIL-12. Because SE inactivated LVs, combination of GLA-SE with ILV/mIL12 was not evaluated.

While intratumoral administration of GLA-SE alone showed no therapeutic benefit against melanoma tumors, the combination of GLA-SE and srRNA/mIL12 efficiently eliminated tumors (S6B and S6C Fig). More strikingly, mice treated with GLA-SE in combination with srRNA/mIL12 that survived their original tumor challenge also rejected a tumor rechallenge, suggesting presence of anti-tumor memory T cells likely developed when the primary tumor regressed. Indeed, a statistically significant increase in melanoma antigen specific CD8 T cells (Fig 6C, *p* = 0.0456 and *p* = 0.005, respectively) was observed in mice treated with GLA-SE + srRNA/mIL12. This significant improvement indicates that the srRNA/mIL12 platform in combination with GLA-SE successfully induced antigen-specific memory T cell generation when the primary tumor was immunologically rejected.

To determine the immune pathways underlying these observations, B16 melanoma tumors that were treated with srRNA/mIL12 alone or in combination with GLA-SE were isolated, and RNA was analyzed using Nanostring transcriptomic analysis. Overall, srRNA/mIL12 treatment induced upregulation of most immunological pathways, indicating that intratumoral IL-12 is highly inflammatory (S7A Fig). Batching performed for dendritic cell function and chemotaxis specifically showed heightened function when tumors were treated with a combination of GLA-SE and IL-12 therapy compared to IL-12 therapy alone. This was also reflected in the dendritic cell and neutrophil scores, which point to infiltration of both innate subsets (S7B Fig). Furthermore, when the most significant genetic changes were ranked for each treatment (in comparison to untreated tumors), a large portion of the most upregulated genes are related to myeloid cell and neutrophil development and chemotaxis (Fig 6D).

Similar studies were performed, and trends observed with ILV/mIL12. However, to avoid deactivating the ILV with the SE adjuvant, we performed a staggered injection where ILV was administered 24 hours prior to GLA-SE being introduced (S7C and S8 Figs).

## Discussion

IL-12 is physiologically produced by dendritic cells and macrophages encountering infectious agents and is the key cytokine linking innate and adaptive immune responses [47]. However, its potency has made recombinant IL-12 therapy challenging to apply successfully in the clinic, particularly in the context of systemic administration [11]. Intratumoral delivery approaches for IL-12 have been proposed and some tested in different indications in the clinic, with varying levels of success. A few examples of strategies recently tested include fusion proteins [48, 49], cell therapies [50, 51], combination with standards of care [52–56] and various gene therapies [27, 57–60].

In this study, we compared two vectored IL-12 delivery platforms that, when administered intratumorally, achieved modulation of the tumor microenvironment (TME) that led to complete regression of injected tumors, partial control of uninjected lesions and sub-optimal protection against re-challenges. Our data indicates that the mIL-12 generated by the delivered transgene drove these therapeutic benefits. ILV delivering an inactive mIL-12 transcript (Fig 2A) had no impact on tumor growth (Fig 2C), while intratumoral ILV/mIL12 induced detectable mIL-12 in sera of treated mice and led to complete tumor regression in majority of mice. Similarly, intratumoral srRNA/GFP did not impact tumor growth (S9 Fig), while srRNA/mIL12 delayed tumor growth (Fig 5C & 5E). Additionally, while our lentiviral vector (LV) is dendritic cell tropic [38], we have demonstrated that both our LV and srRNA formulation can transduce murine cells when administered in vivo [39]. Collectively, these findings demonstrate that despite efficient transgene delivery, ILV and srRNA do not directly kill tumor cells, and the observed anti-tumor efficacy reported here was driven by successful intratumoral generation of mIL-12.

Our ILV is a third-generation lentiviral system that is dendritic cell tropic in humans, which preferentially leads to IL-12 being expressed by the cells that produce and utilize it the most efficiently. While the tropism of ILVs is broader in mice [38], we believe that the primary mechanisms driving therapeutic benefit are recapitulated in mice. Our data demonstrate that ILV/mIL12 promotes infiltration of several antigen-presenting cell populations into the tumor, including dendritic cells. This observation was validated by Nanostring analysis which indicated that several genes associated with myeloid cell chemotaxis were significantly upregulated following ILV/mIL12 treatment (S7 Fig). The combination of upregulated chemotaxis signals with efficient transduction of dendritic cell populations in the tumor provides a promising therapeutic mechanism of action. These activated dendritic cells are then able to stimulate both CD8 T cells [61], in addition to helper CD4 T cells [62] and/or natural killer cells, as the tumor protection resulting from ILV/mIL12 administration was lost when these populations were depleted. Lastly, compared to intratumoral NILV/mIL12, ILV/mIl12 was highly efficacious against not just one but multiple murine tumors (Fig 1G). Our analyses (Fig 1A–1F) show that LV/mIL12 was more efficacious likely because it induced greater expression of mIL-12, leading to greater immune activation (evidenced by IFNγ production). These results suggest that to generate an effective anti-tumor response, a threshold amount of IL-12 in the microenvironment is required to generate effective anti-tumor response. Furthermore, when greater amounts of IL-12 are expressed, there is more extensive leakage from the TME into circulation, which could be a mechanism by which ILV/mIL12 aids in controlling distal tumors as well. This advantage was observed while still maintaining systemic concentrations of IL-12 below toxic levels. Future studies could confirm this by quantifying IL-12 concentrations both in the injected TME, distal tumors and other lymphoid organs.

With our second gene delivery platform, we demonstrated that weekly administration of polyethyleneimine-formulated srRNA/mIL12 resulted in a significant therapeutic benefit as well. Tumor infiltration of immune cells was largely the same in mice treated with ILV/mIL12 and srRNA/mIL12, indicating that both platforms are likely providing similar anti-tumor signals. Interestingly, the amount of IL-12 found in the serum following srRNA/mIL12 intratumoral injection is approximately 10x higher at its peak than after ILV/mIL12 administration, suggesting that the higher expression of IL-12 does not fundamentally change trafficking of infiltrating immune populations into the tumor. ILVs benefited from a longer duration of transgene expression (~3 weeks), which has been shown by others to control establishment and growth of lung tumors in a mouse model over a period of time where IL-12 was detectable in the serum [63]. This is likely why weekly immunizations of srRNA/IL12 were required to achieve the same protection as a single ILV/IL12 injection. On the other hand, the transient expression of srRNA provides additional safety by minimizing unwanted IL-12-associated toxicity.

Despite the highly potent anti-tumor protection that ILV/mIL12 and srRNA/mIL12 provided, the majority of treated mice were not able to mount a sufficient recall response for complete protection upon rechallenge. IL-12 is implicated in effector T cell generation [33], and observations have recently been reported where intratumoral delivery of IL-12 drove rapid apoptotic death of activated tumor-infiltrating effector and memory T cells [64], suggesting high local concentrations of IL-12 may prohibit generation of effective immunological memory. Given that a healthy myeloid compartment is critical in maintenance of memory T cell health, combining local IL-12 therapy with GM-CSF, a stimulator of antigen-presenting cells, could have potentially circumvented the suboptimal generation of immunological memory induced by IL-12 monotherapy. Unfortunately, these investigations did not assess the impact of this combination on long-term protection [65]. Our findings here provide some insight on how activation and mobilization of the myeloid compartment with GLA-SE could overcome suboptimal immunological memory generation as a result of high local concentrations of IL-12.

In an effort to improve both acute and long-term tumor protection, we combined the srRNA/mIL12 treatment with our TLR4 agonist GLA-SE, which is a formulated in a squalene-based oil-in-water emulsion. Lipopolysaccharide (LPS) is a natural potent TLR4 agonist that activates the innate immune compartment, supporting the production of memory CD8 T cell populations [66] and type 1 helper T cell programming [67]. GLA-SE triggers a similar signaling cascade as LPS, with the benefit of having a lower toxicity profile [68]. Indeed, we observed that the combination of GLA-SE with formulated srRNA/mIL12 positively impacted immunological memory formation, allowing mice to reject a second challenge of the parental tumor line despite receiving no further treatments following the rechallenge. In addition to TLR4 signaling, the squalene-based emulsion incorporated into the GLA-SE formulation stimulates inflammasome activation, which we confirmed by Nanostring transcriptional analysis (S8 Fig). Others have shown that GLA-SE induced inflammasome signaling results in IL-18-dependent IFNγ production in memory CD8 T cells and neutrophils [69, 70]. IFNγ-secreting neutrophils were also localized to T cell zones in lymph nodes, allowing them to participate in antigen presentation. Furthermore, this IL-18- IFNγ feedback loop has been shown to drive accelerated proliferation of CD8 memory T cells [71], potentially enhancing a recall response that was strong enough to overcome the detrimental effects of high local IL-12 on immunological memory formation.

Nanostring analysis of tumors treated with the combination of GLA-SE and srRNA/mIL12 revealed that this combination induced the most highly immunologically active tumors that were evaluated. Specifically, tumors treated with the combination therapy had a greater expression increase in genes associated with myeloid and neutrophil chemotaxis and activation than in tumors treated with IL-12 therapy alone. Additionally, TLR-stimulated neutrophils recruit NK cells which then respond to IL-12 by promoting maturation of dendritic cells in the TME [72]. The marriage of TLR4 and inflammasome activation and increased local IL-12 concentration may therefore be a combination that can potently transform the TME into one that is anti-tumor and pro-memory formation.

The TME is naturally hostile for effector immune cells. This work demonstrates that combining immunological signals to tune the TME can synergize to result in a more robust and persistent anti-tumor response. Specifically, the combination of TLR4, inflammasome, and IL-12-driven activation induce a response that protects mice from acute tumor progression, metastasis, and recurrence. Our findings 1) elevate a potential setback for intratumoral IL-12 as a monotherapy (specifically, inability to generate effective immunological memory), 2) provide a potential combination strategy (e.g., with GLA-SE) to overcome this limitation, and 3) propose how GLA-SE-induced TME remodeling potentiated its combination with intratumoral IL-12 to generate effective immunological memory. We believe that the combination of ILV/mIL12 or srRNA/mIL12 with GLA-SE provides an ideal intratumoral treatment for a variety of solid tumors, overcoming the many barriers of previous IL-12 therapies.

## Supporting information

S1 Fig Integrating lentiviral vectors induce greater IL12 expression, leading to superior control in multiple syngeneic tumor models. **(A)** CD11c+HLA-DR+DC-SIGN+ human dendritic cells were transduced with integrating lentiviral vector (ILV) and non-integrating lentiviral vector (NILV) expressing GFP. **(B)** Mice were injected with ILVs or NILVs expressing firefly luciferase subcutaneously at the base of the tail. **(C)** Mice were inoculated subcutaneously with colon carcinoma (CT26), B cell lymphoma (A20), melanoma (B16) or mastocytoma (P815) or orthotopically with mammary carcinoma (4T1), then treated with a single shot of either ILV expressing mIL-12 or NILV expressing mIL12. **(D)** GL261 glioma cells were implanted subcutaneously in the flank of mice that were subsequently treated with ILV/mIL12. Significance was determined by using Mantel-Cox Log-rank testing to compare survival curves of mice treated with NILVs vs ILVs.
(TIFF)

S2 Fig Tumor control following ILV immunization is mediated by IL12. Mice inoculated with either **(A)** B16 melanoma tumors in their footpads or **(B)** CT26 colon carcinoma tumors in their flanks and were treated with a single shot of ILVs expressing mIL12 with or without a fatal mutation in the reverse transcriptase machinery (“RT-mut).
(TIFF)

S3 Fig Depletion of CD4 and CD8 T cells decreased or eliminated survival benefit following intratumoral ILV/mIL12 treatment. Mice inoculated subcutaneously with B16 melanoma tumors in their right flanks were depleted of either **(A)** one or **(B)** two critical immune populations using antibodies against CD8α, CD4 or NK1.1 (to deplete CD8 T cells, CD4 T cells or natural killer cells, respectively) throughout the experiment. Mice were then given a single intratumoral injection of ILV/mIL12 and tumor growth was monitored (*p* < 0.0001). Tumor growth plots represent individual mice.
(TIFF)

S4 Fig Self-replication leads to greater transgene expression than transfection with traditional mRNA. **(A, B)** Baby hamster kidney (BHK) cells were transfected for 24 hours with either self-replicating mRNA or mRNA encoding GFP complexed with Lipofectamine 3000 at varying doses. GFP+ cells were quantified using flow cytometry (*p* < 0.02). **(C)** Mice were injected with srRNA expressing firefly luciferase (1ug) intramuscularly in the right calf muscle. Luciferase expression was monitored over 15 days.
(TIFF)

S5 Fig Weekly immunization of srRNA/IL12 provides better tumor control than a single immunization. **(A, B)** Mice were inoculated with B16 melanoma tumors in their flanks, then treated with either a single shot of ILV/mIL12 or srRNA/mIL12. Tumor growth was monitored thereafter. **(C, D)** Mice were inoculated with B16 melanoma tumors in their flanks, then treated with either a single or weekly shot of srRNA/mIL12. Tumor growth was monitored thereafter.
(TIFF)

S6 Fig The addition of GLA-SE to intratumoral mIL12 imparts protection against tumor rechallenge. **(A)** Mice that rejected an initial challenge of B16 melanoma tumors following ILV/mIL12 treatment were rechallenged with the same parental tumor line. **(B, C)** Mice that rejected an initial challenge of B16 melanoma tumors following srRNA/mIL12 treatment were rechallenged with the same parental tumor line.
(TIFF)

S7 Fig Nanostring profiling identifies genes regulating myeloid and neutrophil chemotaxis and activation as the most upregulated following mIL12+GLA-SE treatment. **(A-C)** Mice were inoculated with B16 melanoma tumors then treated with ILV/mIL12, srRNA/mIL12, GLA-SE or a combination of IL12 therapy with GLA-SE. To avoid inactivation of ILVs with SE adjuvant, injections were staggered such that there was a 24 hour interval between the ILV and GLA-SE doses. Tumors were removed and RNA isolated for Nanostring transcriptomic analysis using their immune oncology gene chip. Genes associated with dendritic cells infiltration and function, neutrophil infiltration and overall chemotaxis were selectively analyzed using hierarchical clustering analysis.
(TIFF)

S8 Fig GLA-SE drives inflammasome activation. **(A, B)** Mice were inoculated with B16 melanoma tumors then treated with ILV/mIL12, srRNA/mIL12, GLA-SE or a combination of IL12 therapy with GLA-SE. To avoid inactivation of ILVs with SE adjuvant, injections were staggered such that there was a 24 hour interval between the ILV and GLA-SE doses. Tumors were removed and RNA isolated for Nanostring transcriptomic analysis using their immune oncology gene chip. Genes associated with inflammasome activity were selectively analyzed using hierarchical clustering analysis.
(TIFF)

S9 Fig Intratumoral administration of srRNA/GFP does not delay tumor growth. **(A, B)** Mice were inoculated with B16 melanoma tumors in their flanks, then treated with either a single shot of ILV/mIL12, srRNA/mIL12 or srRNA/GFP. Tumor growth was monitored thereafter.
(TIFF)

S1 Table Treatment with ILV/mIL12 improves median survival and/or leads to increased tumor regression. Mice were inoculated subcutaneously with colon carcinoma (CT26), B cell lymphoma (A20), melanoma (B16) or mastocytoma (P815) or orthotopically with mammary carcinoma (4T1), then treated with a single shot of either ILV expressing mIL-12 or NILV expressing mIL12. Percentage of mice that completely rejected their tumors are reported if the total percentage of mice surviving after ILV treatment is greater than 50% and therefore median survival could not be calculated. In models where greater than 50% of mice died in all groups, median survival was reported.
(DOCX)

S2 Table Summary of statistical analyses. ANOVA tests with Tukey’s test for multiple comparisons or Mann-Whitney T tests were used to determine statistical significance and performed using Prism 8.0 (GraphPad, La Jolla, CA). Survival statistical significance was determined using a Log-rank test. *P* values equal to or less than 0.05 were considered significant.
(PDF)
