# Supplementary figures and images for: Intratumoral expression of IL-12 from lentiviral or RNA vectors acts synergistically with TLR4 agonist (GLA) to generate anti-tumor immunological memory
Source: PLoS One. 2021 Dec 2;16(12):e0259301. doi: 10.1371/journal.pone.0259301 (PMC8638928; doi:10.1371/journal.pone.0259301)

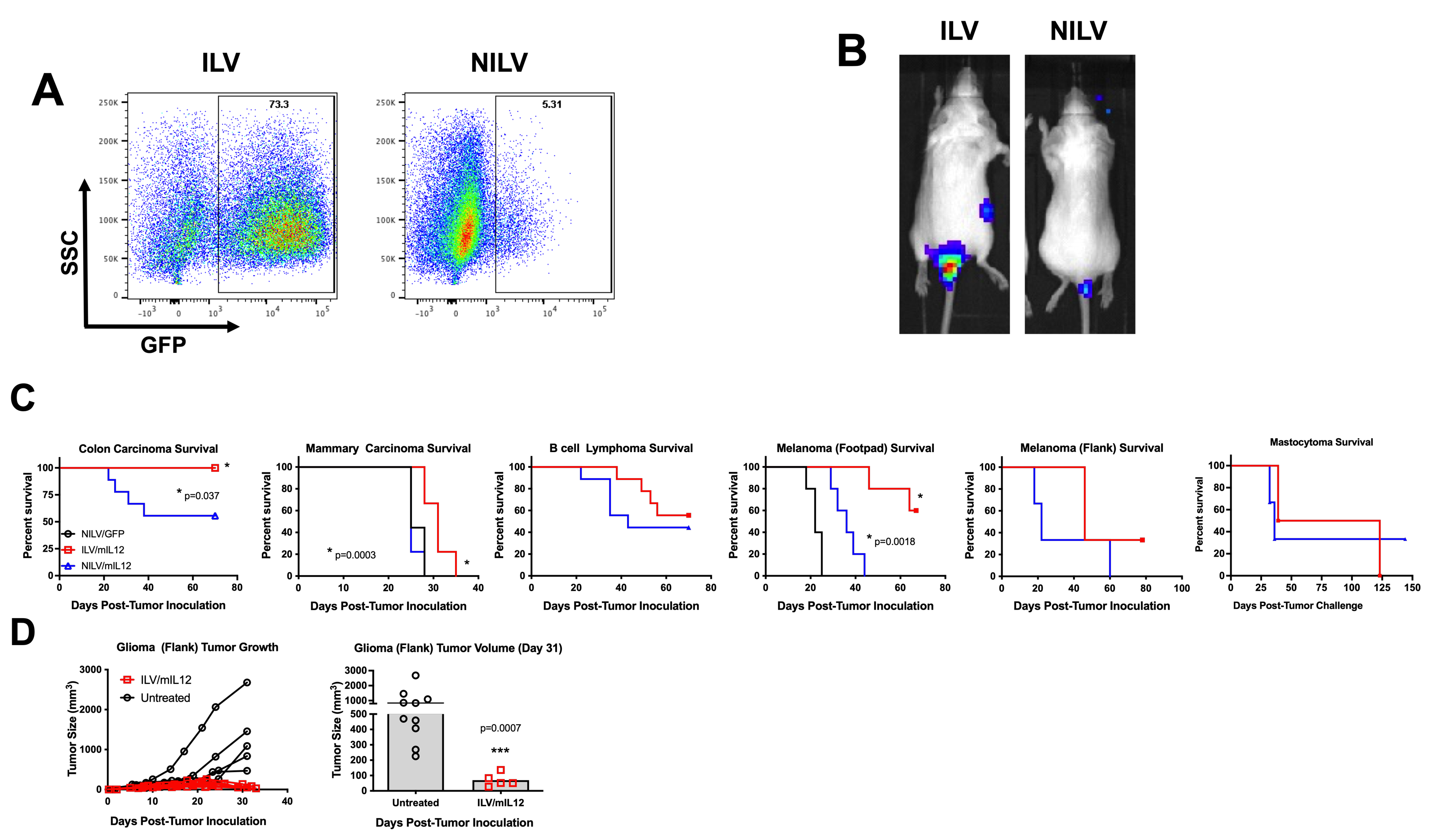

Supplement: S1 Fig — (A) CD11c+HLA-DR+DC-SIGN+ human dendritic cells were transduced with integrating lentiviral vector (ILV) and non-integrating lentiviral vector (NILV) expressing GFP. (B) Mice were injected with ILVs or NILVs expressing firefly luciferase subcutaneously at the base of the tail. (C) Mice were inoculated subcutaneously with colon carcinoma (CT26), B cell lymphoma (A20), melanoma (B16) or mastocytoma (P815) or orthotopically with mammary carcinoma (4T1), then treated with a single shot of either ILV expressing mIL-12 or NILV expressing mIL12. (D) GL261 glioma cells were implanted subcutaneously in the flank of mice that were subsequently treated with ILV/mIL12. Significance was determined by using Mantel-Cox Log-rank testing to compare survival curves of mice treated with NILVs vs ILVs. (TIFF) [file pone.0259301.s001.tiff]

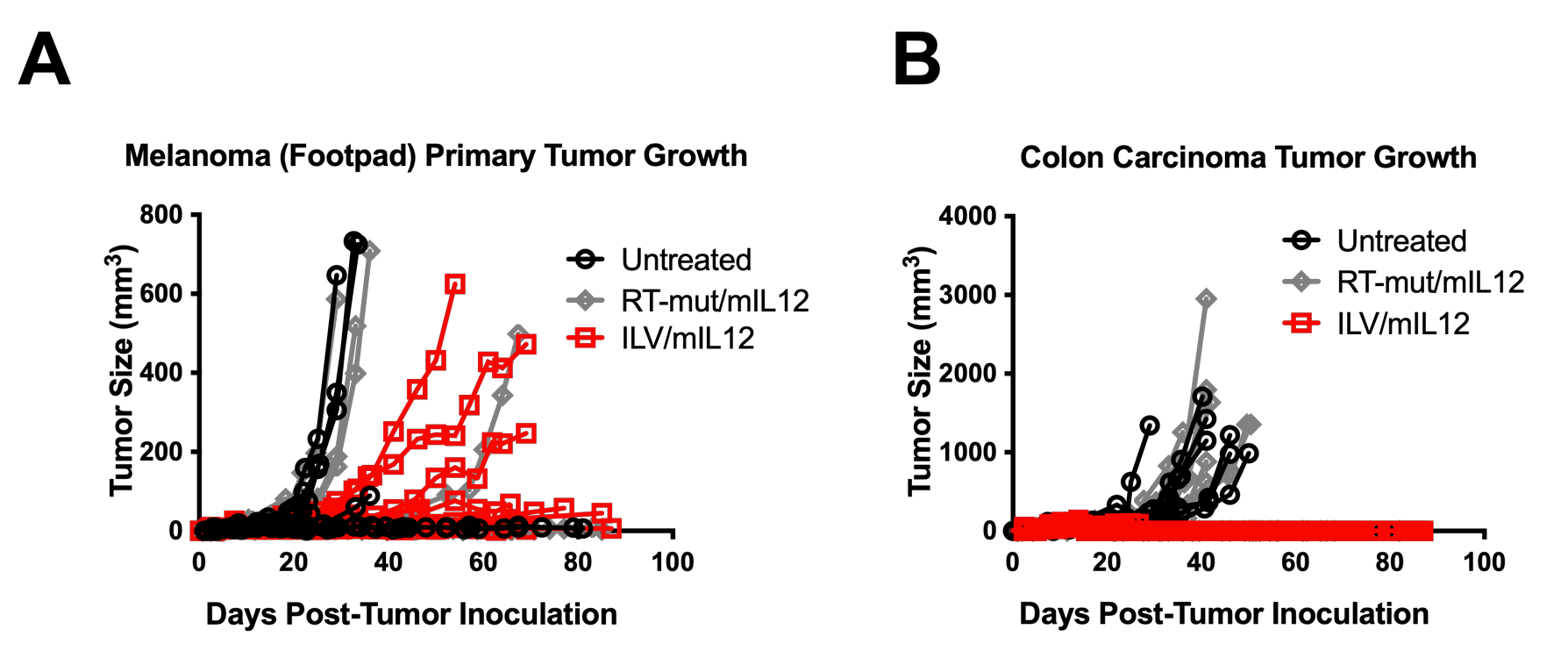

Supplement: S2 Fig — Mice inoculated with either (A) B16 melanoma tumors in their footpads or (B) CT26 colon carcinoma tumors in their flanks and were treated with a single shot of ILVs expressing mIL12 with or without a fatal mutation in the reverse transcriptase machinery (“RT-mut). (TIFF) [file pone.0259301.s002.tiff]

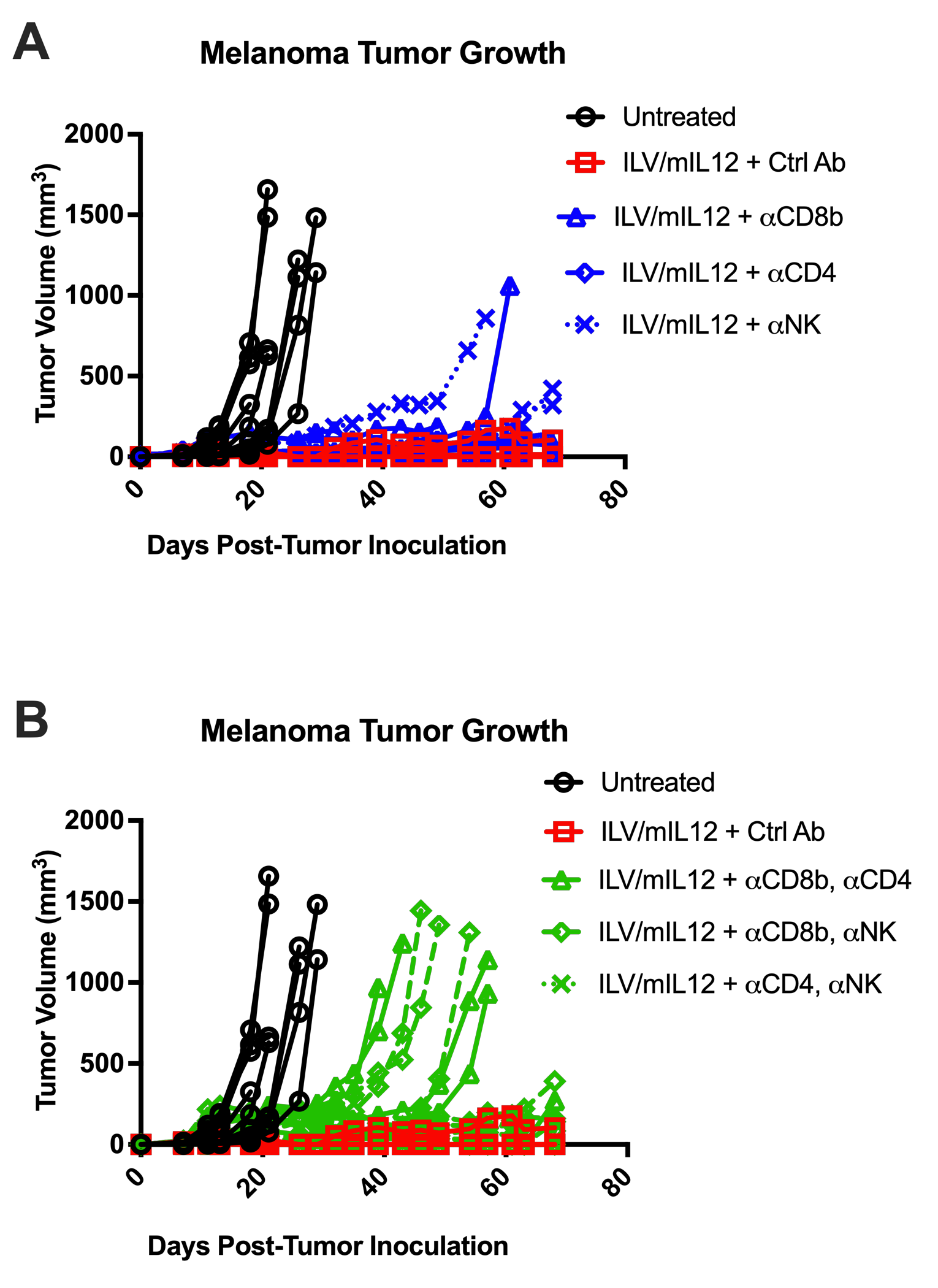

Supplement: S3 Fig — Mice inoculated subcutaneously with B16 melanoma tumors in their right flanks were depleted of either (A) one or (B) two critical immune populations using antibodies against CD8α, CD4 or NK1.1 (to deplete CD8 T cells, CD4 T cells or natural killer cells, respectively) throughout the experiment. Mice were then given a single intratumoral injection of ILV/mIL12 and tumor growth was monitored (p < 0.0001). Tumor growth plots represent individual mice. (TIFF) [file pone.0259301.s003.tiff]

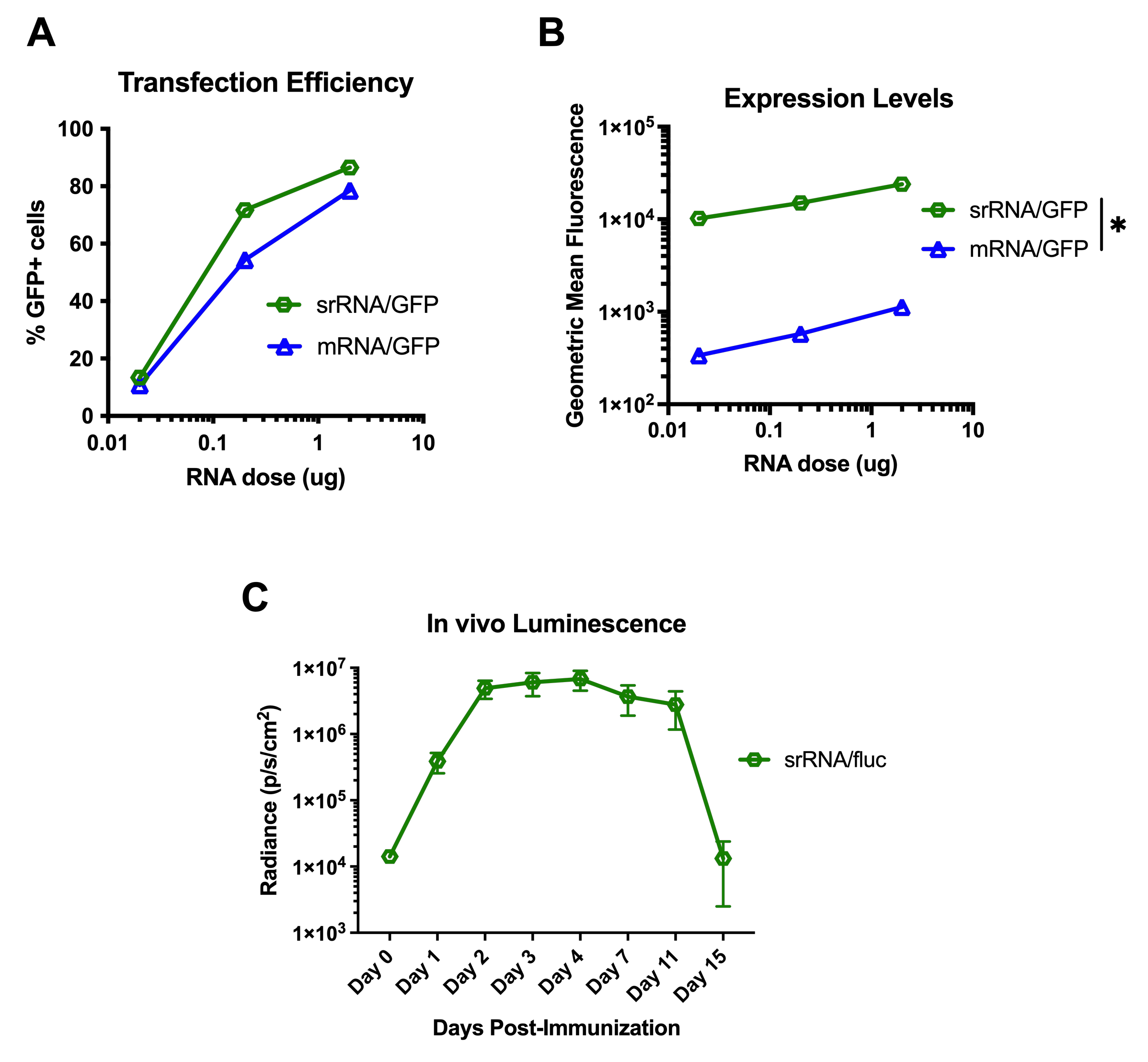

Supplement: S4 Fig — (A, B) Baby hamster kidney (BHK) cells were transfected for 24 hours with either self-replicating mRNA or mRNA encoding GFP complexed with Lipofectamine 3000 at varying doses. GFP+ cells were quantified using flow cytometry (p < 0.02). (C) Mice were injected with srRNA expressing firefly luciferase (1ug) intramuscularly in the right calf muscle. Luciferase expression was monitored over 15 days. (TIFF) [file pone.0259301.s004.tiff]

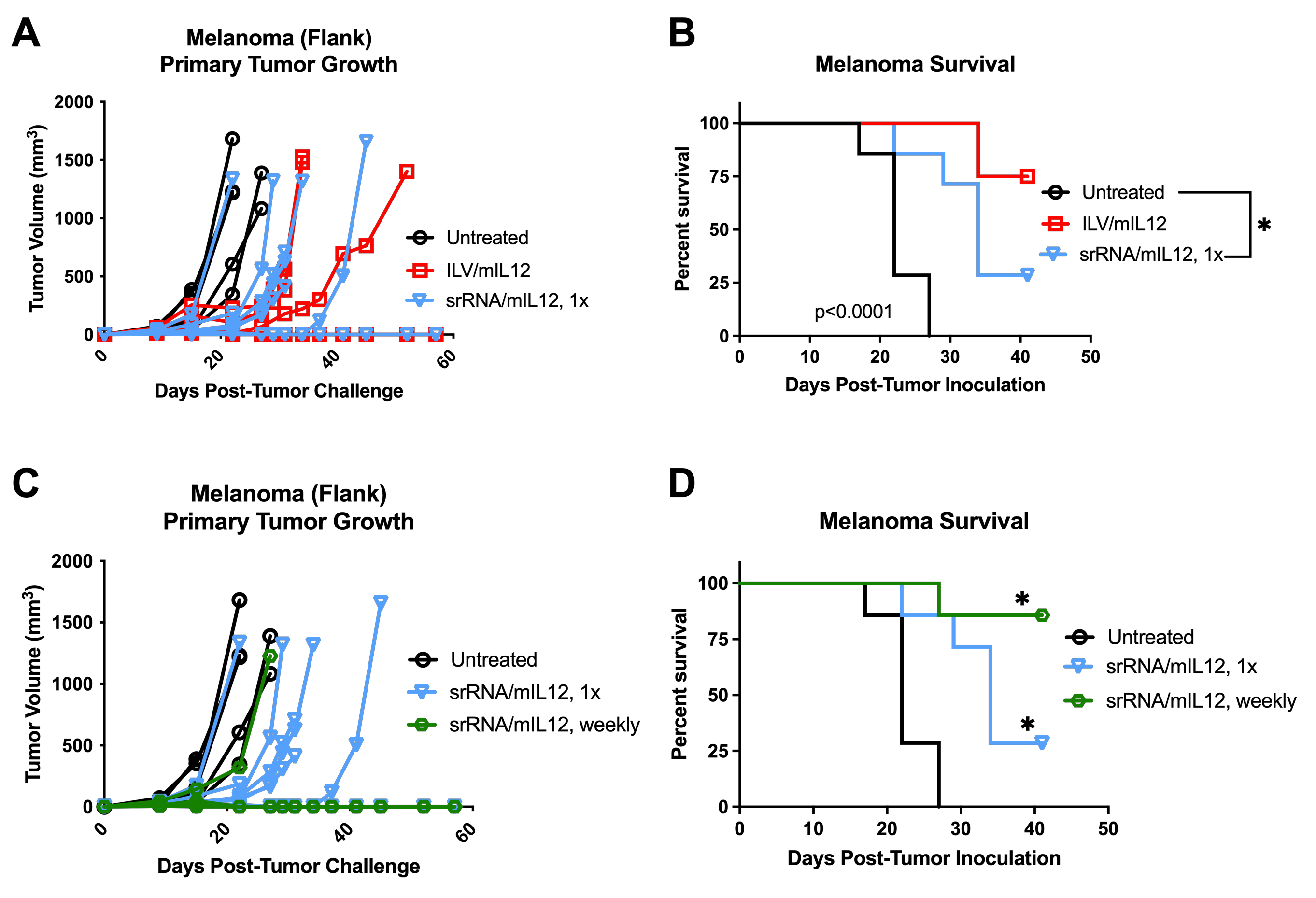

Supplement: S5 Fig — (A, B) Mice were inoculated with B16 melanoma tumors in their flanks, then treated with either a single shot of ILV/mIL12 or srRNA/mIL12. Tumor growth was monitored thereafter. (C, D) Mice were inoculated with B16 melanoma tumors in their flanks, then treated with either a single or weekly shot of srRNA/mIL12. Tumor growth was monitored thereafter. (TIFF) [file pone.0259301.s005.tiff]

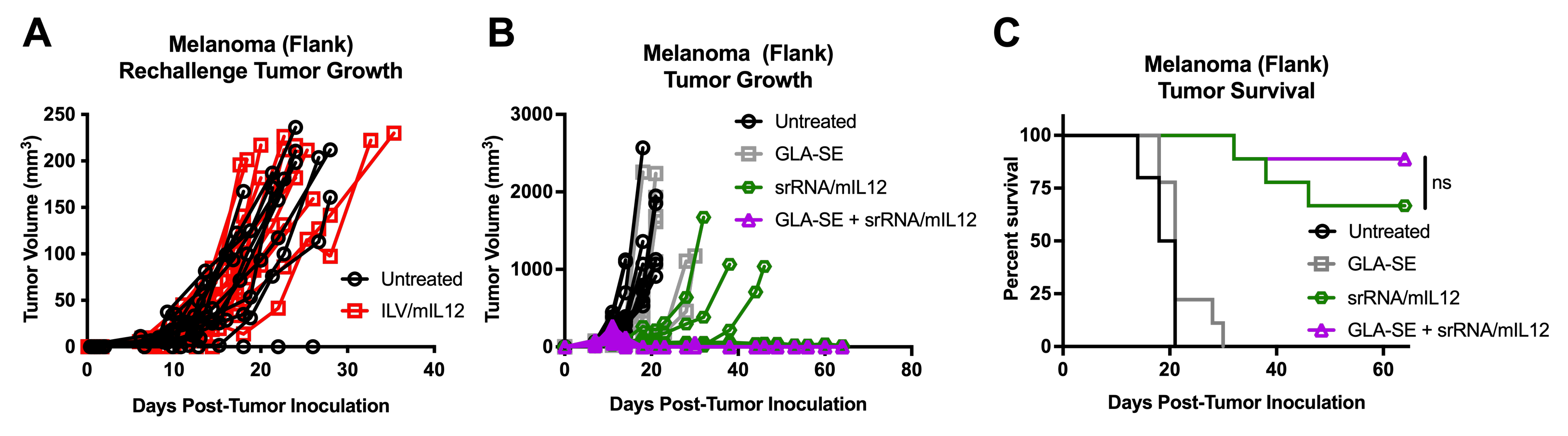

Supplement: S6 Fig — (A) Mice that rejected an initial challenge of B16 melanoma tumors following ILV/mIL12 treatment were rechallenged with the same parental tumor line. (B, C) Mice that rejected an initial challenge of B16 melanoma tumors following srRNA/mIL12 treatment were rechallenged with the same parental tumor line. (TIFF) [file pone.0259301.s006.tiff]

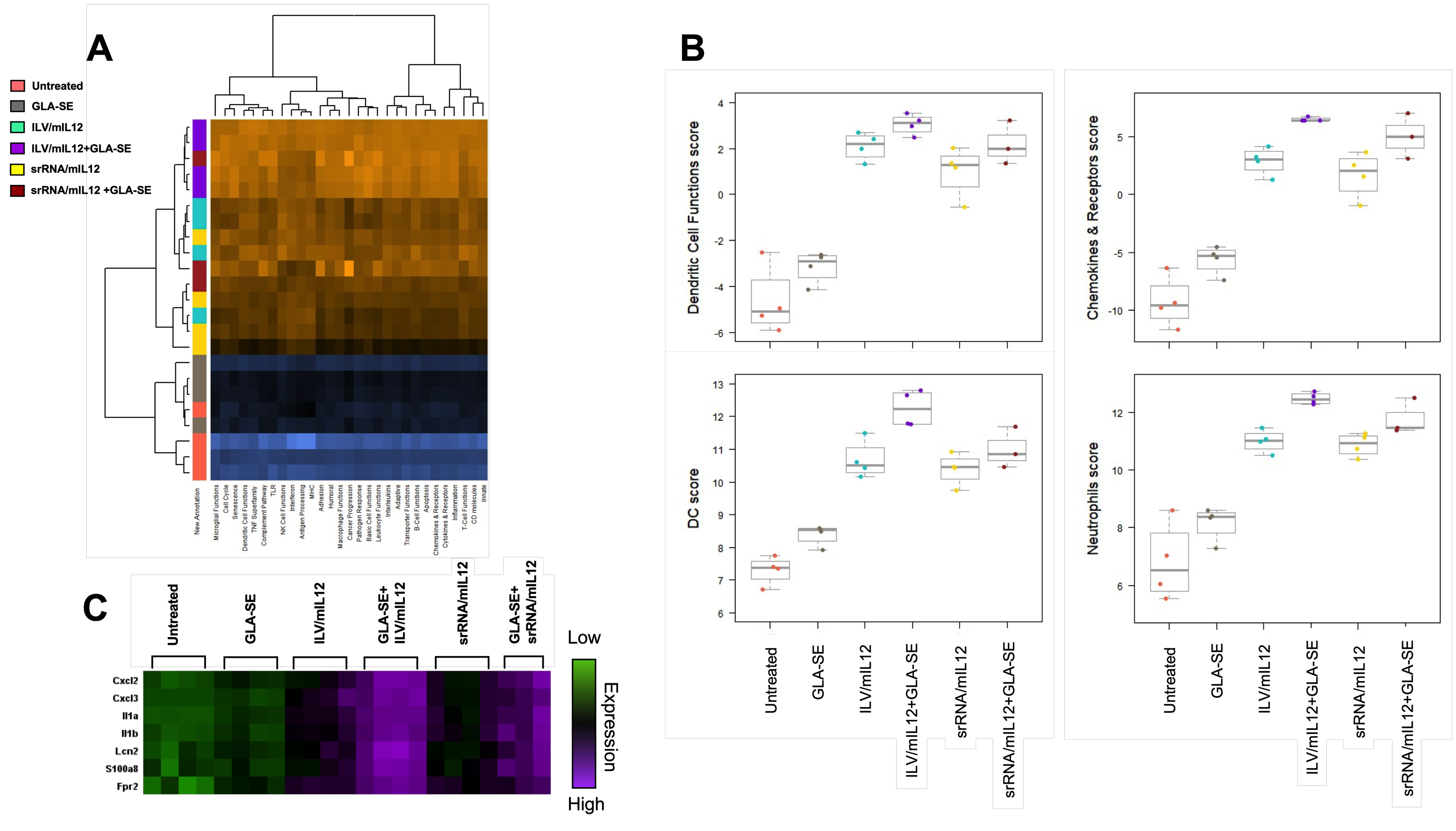

Supplement: S7 Fig — (A-C) Mice were inoculated with B16 melanoma tumors then treated with ILV/mIL12, srRNA/mIL12, GLA-SE or a combination of IL12 therapy with GLA-SE. To avoid inactivation of ILVs with SE adjuvant, injections were staggered such that there was a 24 hour interval between the ILV and GLA-SE doses. Tumors were removed and RNA isolated for Nanostring transcriptomic analysis using their immune oncology gene chip. Genes associated with dendritic cells infiltration and function, neutrophil infiltration and overall chemotaxis were selectively analyzed using hierarchical clustering analysis. (TIFF) [file pone.0259301.s007.tiff]

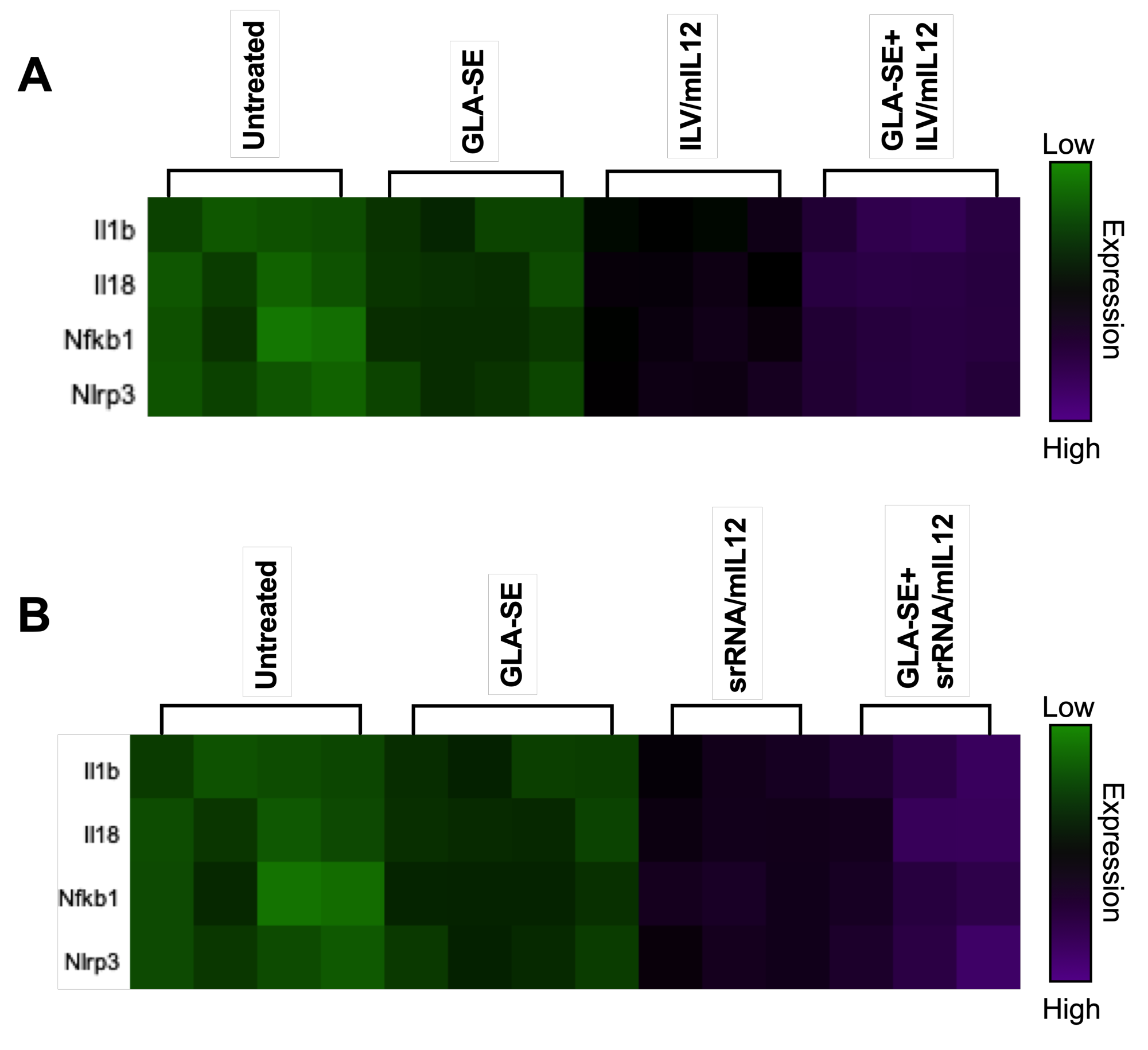

Supplement: S8 Fig — (A, B) Mice were inoculated with B16 melanoma tumors then treated with ILV/mIL12, srRNA/mIL12, GLA-SE or a combination of IL12 therapy with GLA-SE. To avoid inactivation of ILVs with SE adjuvant, injections were staggered such that there was a 24 hour interval between the ILV and GLA-SE doses. Tumors were removed and RNA isolated for Nanostring transcriptomic analysis using their immune oncology gene chip. Genes associated with inflammasome activity were selectively analyzed using hierarchical clustering analysis. (TIFF) [file pone.0259301.s008.tiff]

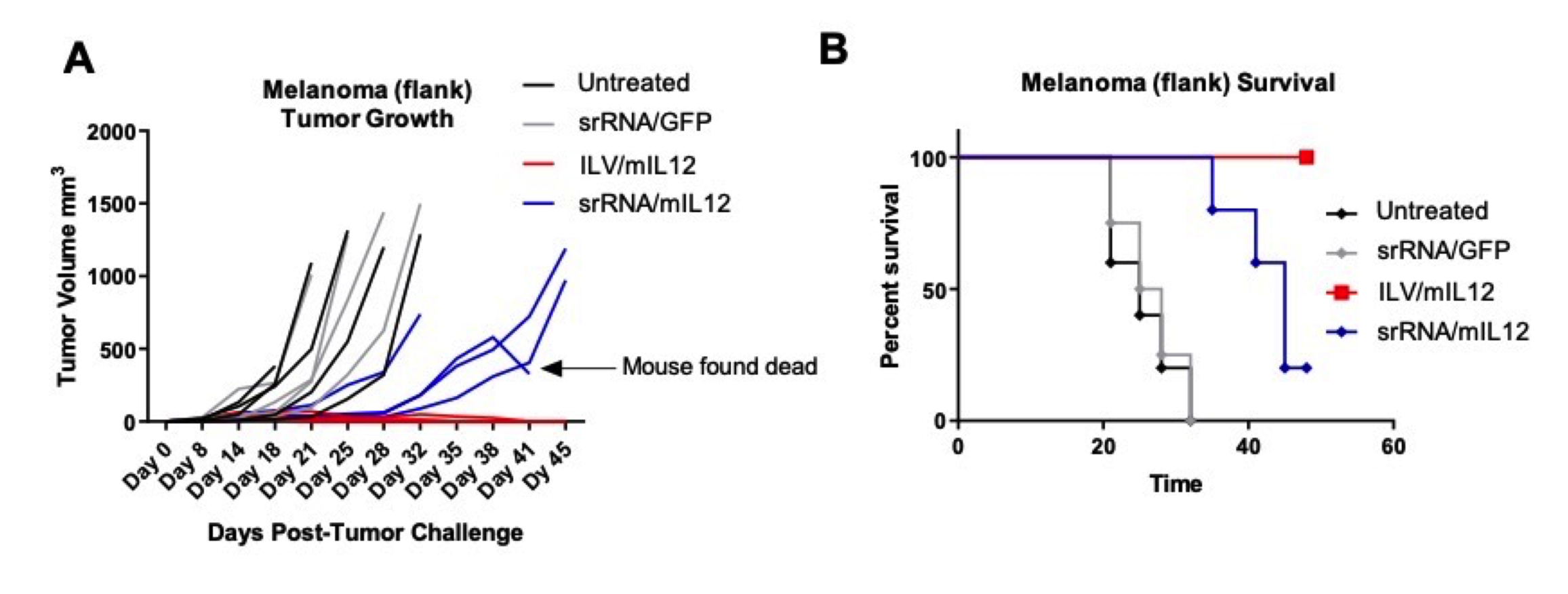

Supplement: S9 Fig — (A, B) Mice were inoculated with B16 melanoma tumors in their flanks, then treated with either a single shot of ILV/mIL12, srRNA/mIL12 or srRNA/GFP. Tumor growth was monitored thereafter. (TIFF) [file pone.0259301.s009.tiff]
