# Supplementary material for: Intratumoral expression of IL-12 from lentiviral or RNA vectors acts synergistically with TLR4 agonist (GLA) to generate anti-tumor immunological memory
Source: PLoS One. 2021 Dec 2;16(12):e0259301. doi: 10.1371/journal.pone.0259301 (PMC8638928; doi:10.1371/journal.pone.0259301)
Supplement: S1 Table — Mice were inoculated subcutaneously with colon carcinoma (CT26), B cell lymphoma (A20), melanoma (B16) or mastocytoma (P815) or orthotopically with mammary carcinoma (4T1), then treated with a single shot of either ILV expressing mIL-12 or NILV expressing mIL12. Percentage of mice that completely rejected their tumors are reported if the total percentage of mice surviving after ILV treatment is greater than 50% and therefore median survival could not be calculated. In models where greater than 50% of mice died in all groups, median survival was reported. (DOCX) [file pone.0259301.s010.docx]

**S1 Table. Treatment with ILV/mIL12 improves median survival and/or leads to increased tumor regression.** Mice were inoculated subcutaneously with colon carcinoma (CT26), B cell lymphoma (A20), melanoma (B16) or mastocytoma (P815) or orthotopically with mammary carcinoma (4T1), then treated with a single shot of either ILV expressing mIL-12 or NILV expressing mIL12. Percentage of mice that completely rejected their tumors are reported if the total percentage of mice surviving after ILV treatment is greater than 50% and therefore median survival could not be calculated. In models where greater than 50% of mice died in all groups, median survival was reported.
