# Supplementary material for: Intratumoral expression of IL-12 from lentiviral or RNA vectors acts synergistically with TLR4 agonist (GLA) to generate anti-tumor immunological memory
Source: PLoS One. 2021 Dec 2;16(12):e0259301. doi: 10.1371/journal.pone.0259301 (PMC8638928; doi:10.1371/journal.pone.0259301)
Supplement: S2 Table — ANOVA tests with Tukey’s test for multiple comparisons or Mann-Whitney T tests were used to determine statistical significance and performed using Prism 8.0 (GraphPad, La Jolla, CA). Survival statistical significance was determined using a Log-rank test. P values equal to or less than 0.05 were considered significant. (PDF) [file pone.0259301.s011.pdf]

Figure	Test	Comparison	P Value	Significant?	# Animals
Figure 1A	1-way ANOVA+Tukey	NILV/GFP vs ILV/GFP	<0.0001	Y	N/A
Figure 1B	1-way ANOVA+Tukey	NILV/GFP vs ILV/GFP	<0.0001	Y	N/A
Figure 1C	1-way ANOVA+Tukey per time point	NILV/GFP vs ILV/GFP, Day 3	0.08	N	n = 6, total = 12
Figure 1C	1-way ANOVA+Tukey per time point	NILV/GFP vs ILV/GFP, Day 4	0.006	Y	n = 6, total = 12
Figure 1C	1-way ANOVA+Tukey per time point	NILV/GFP vs ILV/GFP, Day 7	<0.0001	Y	n = 6, total = 12
Figure 1E	1-way ANOVA+Tukey per time point	NILV/GFP vs ILV/GFP, Day 6	0.13	N	n = 4-7, total = 18
Figure 1E	1-way ANOVA+Tukey per time point	NILV/GFP vs ILV/GFP, Day 15	0.02	Y	n = 4-7, total = 18
Figure 1F	1-way ANOVA+Tukey per time point	NILV/GFP vs ILV/GFP, Day 6	0.02	Y	n = 4-7, total = 18
Figure 1F	1-way ANOVA+Tukey per time point	NILV/GFP vs ILV/GFP, Day 15	<0.0001	Y	n = 4-7, total = 18
Figure 1F	1-way ANOVA+Tukey per time point	NILV/GFP vs ILV/GFP, Day 21	0.001	Y	n = 4-7, total = 18
Figure 2A	1-way ANOVA+Tukey per time point	NILV/GFP vs ILV/GFP, Day 6	0.13	N	n = 4-7, total = 18
Figure 2A	1-way ANOVA+Tukey per time point	NILV/GFP vs ILV/GFP, Day 15	0.02	Y	n = 4-7, total= 18
Figure 2B	1-way ANOVA+Tukey per time point	NILV/GFP vs ILV/GFP, Day 6	0.02	Y	n = 4-7, total = 18
Figure 2B	1-way ANOVA+Tukey per time point	NILV/GFP vs ILV/GFP, Day 15	<0.0001	Y	n = 4-7, total = 18
Figure 2B	1-way ANOVA+Tukey per time point	NILV/GFP vs ILV/GFP, Day 21	0.001	Y	n = 4-7, total = 18
Figure 2D	Log rank (Mantel-Cox)	Untreated vs RT-mut/mIL12	0.91	N	n = 8, total = 24
Figure 2D	Log rank (Mantel-Cox)	RT-mut/mIL12 vs ILV/mIL12	0.0002	Y	n = 8, total = 24
Figure 3C	Log rank (Mantel-Cox)	Untreated vs ILV/mIL12	0.29	N	n = 8, total = 16
Figure 3F	Log rank (Mantel-Cox)	Untreated vs ILV/mIL12	<0.0001	Y	n = 8, total = 16
Figure 3I	Log rank (Mantel-Cox)	Untreated vs ILV/mIL12	0.009	Y	n = 9, total = 18
Figure 3K	Mann-Whitney T test	Untreated vs ILV/mIL12	0.0003	Y	n = 7-8, total = 15
Figure 4A	Log rank (Mantel-Cox)	Cntrl Ab vs aCD8	0.37	N	n = 8-9, total = 30
Figure 4A	Log rank (Mantel-Cox)	Cntrl Ab vs aCD4	0.17	N	n = 8-9, total = 30
Figure 4A	Log rank (Mantel-Cox)	Cntrl Ab vs aNK	0.39	N	n = 8-9, total = 30
Figure 4B	Log rank (Mantel-Cox)	Cntrl Ab vs aCD8, CD4	<0.0001	Y	n = 5-10,total = 32

Figure 4B	Log rank (Mantel-Cox)	Cntrl Ab vs aCD8, aNK	0.002	Y	n = 5-10,total = 32
Figure 4B	Log rank (Mantel-Cox)	Cntrl Ab vs CD4, aNK	0.12	N	n = 5-10,total = 32
Figure 5D	Log rank (Mantel-Cox)	Untreated vs srRNA/mIL12	<0.0001	Y	n = 9-10,total = 19
Figure 5F	Log rank (Mantel-Cox)	Untreated vs srRNA/mIL12	<0.0001	Y	n = 8-9,total = 17
Figure 5H	1-way ANOVA+Tukey	Untreated vs srRNA/mIL12, IT	0.0016	Y	n = 8-10,total = 28
Figure 5H	1-way ANOVA+Tukey	Untreated vs srRNA/mIL12, SC	<0.0001	Y	n = 8-10,total = 28
Figure 6B	Log rank (Mantel-Cox)	srRNA/mIL12 vs GLA-SE+srRNA/mIL12	0.007	Y	n = 9-10,total = 36
Figure 6C	1-way ANOVA+Tukey	mTRP1, Untreated vs srRNA/mIL12	0.85	N	n = 9-10,total = 36
Figure 6C	1-way ANOVA+Tukey	mTRP1, Untreated vs GLA-SE+srRNA/mIL12	0.04	Y	n = 9-10,total = 36
Figure 6C	1-way ANOVA+Tukey	gp100, Untreated vs srRNA/mIL12	0.43	N	n = 9-10,total = 36
Figure 6C	1-way ANOVA+Tukey	gp100, Untreated vs GLA-SE+srRNA/mIL12	0.005	Y	n = 9-10,total = 36
SuppFig 1C	Log rank (Mantel-Cox)	NILV/mIL12 vs ILV/mIL12, colon	0.037	Y	n = 6, total = 12
SuppFig 1C	Log rank (Mantel-Cox)	NILV/mIL12 vs ILV/mIL12, mammary	0.0003	Y	n = 6, total = 12
SuppFig 1C	Log rank (Mantel-Cox)	NILV/mIL12 vs ILV/mIL12, B cell	0.38	N	n = 6, total = 12
SuppFig 1C	Log rank (Mantel-Cox)	NILV/mIL12 vs ILV/mIL12, melanoma (footpad)	0.0018	Y	n = 6, total = 12
SuppFig 1C	Log rank (Mantel-Cox)	NILV/mIL12 vs ILV/mIL12, melanoma (flank)	0.27	N	n = 6, total = 12
SuppFig 1C	Log rank (Mantel-Cox)	NILV/mIL12 vs ILV/mIL12, mastocytoma	0.95	N	n = 6, total = 12
SuppFig 1D	Mann-Whitney	Untreated vs ILV/mIL12	0.0007	Y	n = 5-10, total = 15
SuppFig 4B	Unpaired T test	srRNA/GFP vs mRNA/GFP	0.02	Y	n = 5-10, total = 32
SuppFig 5B	Log rank (Mantel-Cox)	Untreated vs srRNA/mIL12, 1x	<0.0001	Y	n = 6-8, total = 21
SuppFig 5D	Log rank (Mantel-Cox)	Untreated vs srRNA/mIL12, 1x	0.002	Y	n = 9-10,total = 19
SuppFig5D	Log rank (Mantel-Cox)	Untreated vs srRNA/mIL12, weekly	0.0007	Y	n = 9-10,total = 19
SuppFig6C	Log rank (Mantel-Cox)	srRNA/mIL12 vs GLA-SE+srRNA/mIL12	0.29	N	n = 9-10,total = 36
